# Cyclin-dependent kinases and rare developmental disorders

**DOI:** 10.1186/s13023-020-01472-y

**Published:** 2020-08-06

**Authors:** Pierre Colas

**Affiliations:** Laboratory of Integrative Biology of Marine Models, Station Biologique de Roscoff, Sorbonne Université / CNRS, Roscoff, France

**Keywords:** CDKs, Cyclins, Developmental disorders, Regulatory networks, Interaction profiling

## Abstract

Extensive studies in the past 30 years have established that cyclin-dependent kinases (CDKs) exert many diverse, important functions in a number of molecular and cellular processes that are at play during development. Not surprisingly, mutations affecting CDKs or their activating cyclin subunits have been involved in a variety of rare human developmental disorders. These recent findings are reviewed herein, giving a particular attention to the discovered mutations and their demonstrated or hypothesized functional consequences, which can account for pathological human phenotypes. The review highlights novel, important CDK or cyclin functions that were unveiled by their association with human disorders, and it discusses the shortcomings of mouse models to reveal some of these functions. It explains how human genetics can be used in combination with proteome-scale interaction databases to loom regulatory networks around CDKs and cyclins. Finally, it advocates the use of these networks to profile pathogenic CDK or cyclin variants, in order to gain knowledge on protein function and on pathogenic mechanisms.

## Introduction

Cyclin-dependent kinases (CDKs) form a family of 20 serine/threonine protein kinases that exert pivotal functions in fundamental cellular and molecular processes, such as cell division, migration, senescence, death, gene transcription, mRNA splicing, metabolism, and other important mechanisms (reviewed in [1, 2]. As indicated by their name and in addition to post-translational modifications, they require a physical association with a cyclin partner to become catalytically active and able to phosphorylate their protein substrates. Over 30 cyclins have been identified in humans, on the basis of the presence of a cyclin box domain that is responsible for binding and activating CDKs. Functional and phylogenetic studies distinguish 3 subfamilies of CDK and cyclin proteins (cell cycle, transcriptional, atypical) that form combinatorial interactions mostly within each subfamily [3]. Overexpression and/or dysfunction of CDKs or cyclins have been reported in a very large number of human cancers and other diverse pathologies. These protein kinases are thus considered as valuable therapeutic targets for drug development. A first set of CDK selective inhibitors have been approved recently against hormone-dependent/HER2-negative breast cancers, and they hold promises against other solid tumors [4].

The first involvement of a CDK in a rare disease, familial melanoma, was reported more than 25 years ago, with the discovery of pathogenic mutations in the *CDKN2* gene that codes for an inhibitor of CDK4, soon followed by the discovery of mutations in the *CDK4* gene itself (reviewed in [5]). CDK4 stood splendidly isolated for a long time, with no further CDK or cyclin involved in any other rare disorder. Over the past 12 years, methodological advances in human genomics and major efforts invested in the identification of the genetic causes of human diseases (reviewed in [6]) have allowed a remarkable series of discoveries linking 6 different CDKs and 4 different cyclins to rare developmental disorders (Table 1). In view of the crucial roles played by CDKs and cyclins in the regulation of many cellular processes at play during development, their involvement in a variety of human disorders could be expected. The purpose of this article is to provide a review of these recent discoveries, highlight their surprising contributions to our knowledge on CDK / cyclin functions, explain how they can help to chart regulatory networks, and plead for the interaction profiling of certain types of pathogenic variants to better understand CDK / cyclin functions and to elucidate disease mechanisms.

**Table 1:** Overview of CDKs and cyclins involved in developmental disorders

Gene	CDK or cyclin subfamily; CDK or cyclin main partner(s)	Disease (phenotype MIM number)	Inheritance	Mutations	Functional impact (mechanism)	References
*CDK5*	Atypical; p25/p35	Lissencephaly 7 with cerebellar hypoplasia (616342)	Autosomal Recessive	Splice site	Loss of function (NMD)	[7]
*CDK6*	Cell Cycle; D-type cyclins (*CCND1,2,3*)	Primary microcephaly 12 (616080)	Autosomal Recessive	Missense	Loss of function (misslocalization)	[8]
*CDK8*	Transcriptional; Cyclin C (*CCNC*)	Intellectual developmental disorder with hypotonia and behavioral abnormalities (618748)	Autosomal Dominant	Missense in kinase domain	Loss of function (dominant negative effects?)	[9]
*CDK10*	Transcriptional; Cyclin M (*CCNQ*)	Al Kaissi syndrome (617694)	Autosomal Recessive	- Splice sites - Deletions - Frameshift	Loss of function (NMD) Misslocalization?	[10, 11]
*CDK13*	Transcriptional; Cyclin K (*CCNK*)	Congenital heart defects, dysmorphic facial features, intellectual developmental disorder (617360)	Autosomal Dominant	- Missense - Splice site - Nonsense - Frameshift	Loss of function (dominant negative effects? haploinsufficiency?)	[12–20]
*CDK19*	Transcriptional; Cyclin C (*CCNC*)	Bilateral congenital retinal folds, microcephaly and mild mental retardation (unavailable)	Autosomal Dominant	Chromosomal pericentric inversion	Haploinsufficiency	[21]
*CCND2*	Cell Cycle; CDK6	Megalencephaly-polymicrogyria-polydactyly-hydrocephalus syndrome 3 (615938)	Autosomal Dominant	- Missense - Nonsense	Gain of function (stabilization)	[22]
*CCNK*	Transcriptional; CDK12 and CDK13	Intellectual developmental disorder with hypertelorism and distinctive facies (618147)	Autosomal Dominant	- Deletion - Missense	Loss of function (haploinsufficiency)	[23]
*CCNO*	Atypical; unknown	Primary ciliary dyskinesia 29 (615872)	Autosomal Recessive	-Frameshift - Missense - Nonsense	Loss of function (truncations, 1 missense)	[24–28]
*CCNQ*	Transcriptional; CDK10	STAR syndrome (300707)	X-linked Dominant	- Deletions - Splice site - Frameshift	Loss of function	[29–35]

Source: Online Mendelian Inheritance in Man database (www.omim.org).

### CDK5 and lissencephaly with cerebellar hypoplasia

Lissencephalies are hereditary brain malformations characterized by the absence or paucity of cerebral convolutions, causing the brain surface to appear unusually smooth. They form a heterogeneous group of disorders with different cortical morphologies and various associated malformations, caused by mutations on at least 19 genes mostly coding for microtubule structural proteins or microtubule-associated proteins (reviewed in [36]). Ten children coming from a highly consanguineous family and presenting a rare form of lissencephaly with cerebellar hypoplasia (LCH) were investigated [7]. In addition to an extreme form of LCH, they presented an agenesis of the corpus callosum, microcephaly, severe neurological defects, thick skin, permanently fixed joints and a constellation of facial dysmorphisms. They all died between 2 days to 3 months after birth from respiratory failure. All affected children presented a homozygous point mutation on the splice donor site in intron 8 of *CDK5*, which was not detected in 200 ethnically matched control individuals. The involvement of this mutation in the LCH was further confirmed by a whole-exome sequencing on one of the patients, in which the only homozygous variant that segregated with the disease was *CDK5*. In contrast with control fibroblasts, patient-derived fibroblasts contain undetectable CDK5 mRNA and protein expression levels, which strongly suggests nonsense-mediated decay (NMD) of the mRNA. Even if a protein were expressed, the C-terminal truncation caused by the mutation would be expected to severely compromise the structure of the kinase activation loop, which is required for CDK5 binding to its activating proteins p35/25 [37]. In accordance with this prediction, this C-terminally truncated form of CDK5 fails to complement a deletion of the *PHO85* gene in yeast, whose growth on galactose is rescued by wild-type CDK5. It is thus safe to conclude that this mutation causes a total loss-of-function of CDK5 [7].

CDK5 presents unique features among the CDK family. It is activated by p35 and p39, which exhibit no sequence similarity with cyclins but adopt a three-dimensional structure similar to that of a cyclin box. CDK5 is the only CDK that is active in post-mitotic neurons, where it exerts essential functions such as survival, memory formation, pain signaling. As observed in a number of mice knockout studies, it is essential for normal brain development where it controls neuronal migration, axonal guidance and synaptic plasticity (reviewed in [38]). Hence, the loss-of-function of CDK5 readily accounts for the dramatic clinical condition of the above-described patients. Interestingly, heterozygous silent and intronic mutations in *CDK5* have also been detected in individuals affected by non-syndromic intellectual disability (NS-ID) [39]. The functional consequences have not been fully explored, but a resulting hypothetic partial haploinsufficiency might account for NS-ID, in view of the dramatic effects caused by a total *CDK5* loss-of-function.

### CDK6 and primary microcephaly

Autosomal recessive primary microcephaly (MCPH) is a neurodevelopmental disorder characterized by reduced head circumference and cerebral cortex size, and nonprogressive, variable intellectual disability. So far, 25 genes have been associated with MCPH (reviewed in [40]). Ten children spanning three generations of a consanguineous family presented MCPH with sloping forehead and mild intellectual disability. After exclusion of all known MCPH loci, 49 candidate genes were scrutinized and a single nucleotide mutation was detected in exon 5 of *CDK6*. It perfectly segregated with the MCPH within the investigated family and was neither detected in hundreds of control individuals nor in databases totaling 6500 exomes. The mutation substitutes alanine 197 for a threonine, which abrogates the localization of CDK6 on centrosomes during mitosis, disorganizes the mitotic spindles and affects nuclear morphology. Patient-derived fibroblasts present a lower growth rate and a higher rate of apoptosis than control cells [8]. It is worth noting that other genes involved in MCPH play important roles in mitotic spindle orientation and in centrosome assembly and separation, which underscores the importance of this organelle in neurodevelopment [40].

CDK6 is a key player in the control of the transition from the G1 to the S phase of the cell cycle. Akin to CDK4, it associates with D-type cyclins and phosphorylates members of the retinoblastoma (Rb) tumor suppressor family. This results in the release of E2F transcription factors that control the expression of genes involved in cell cycle progression and DNA replication (reviewed in [41]). The effects produced by the MCPH-causing mutation are presently unknown. The substituted residue is located in a loop that is distant from the catalytic center and from the cyclin or the INK inhibitor binding interfaces. Since patient fibroblasts and *CDK6*-knockdown cells present similar defects, the mislocalization of CDK6 is suspected to cause a loss of function that is not compensated by its close paralog CDK4 [8]. Interestingly, this pathogenic mutation might only cause a loss of the centrosome-associated functions of CDK6, while preserving its transcriptional functions that are particularly important in hematopoiesis [41]. In support of this hypothesis, no immunity defect has been reported in these MCPH patients [8].

### Cyclin D2 and megalencephaly-polymicrogyria-polydactyly-hydrocephalus syndrome

Megalencephaly-polymicrogyria-polydactyly-hydrocephaly (MPPH) syndromes are brain and head overgrowth disorders associated with distal limb malformations, inducing delayed development and intellectual disability. Germline mutations in the *CCND2* gene coding for cyclin D2 were detected by whole-exome sequencing in three MPPH patients who did not present mutations in genes previously involved in the syndrome. De novo heterozygous *CCND2* mutations were then detected in 9 additional MPPH cases. Nine of the 12 cases present missense mutations on threonine 280 or proline 281, 2 cases present a mutation that produces a stop codon after amino acid 270, and 1 case contains a missense mutation affecting valine 284. All 3 substituted residues are highly conserved, and the truncated 270 amino acid protein is likely to be expressed since the mutation occurs in the final exon [22]. More recently, two additional individuals were described, presenting a missense mutation on threonine 280 and proline 281, and an expanded cerebral and cardiac phenotype, respectively [42, 43].

Cyclin D2 was already known to be phosphorylated on threonine 280 by GSK-3β and p38MAPK, which triggers its degradation by the proteasome as demonstrated by the resistance of a T280A cyclin D2 mutant to ubiquitin-dependent degradation [44]. All mutated forms listed above were found to be stabilized and to accumulate when expressed in a human cell line, in contrast to wild-type cyclin D2 [22]. Hence, mutations in *CCND2* causing MPPH induce a gain-of-function of cyclin D2. The importance of cyclin D2 T280 and P281 residues for the development of ventricular and subventricular zones (VZ and SVZ) in mouse embryos was examined by electroporating *CCND2* expression constructs in utero. These elegant experiments showed a strong association between the expression of *CCND2* mutants found in MPPH and actively dividing cells, contrary to the expression of wild-type *CCND2*. This indicates that stabilized cyclin D2 supports abnormal proliferation, which yields an increased population of neural progenitors (radial glial cells and intermediate progenitor cells) that could account for the megalencephaly observed in MPPH [22].

The opposite phenotypes produced by CDK6 loss-of-function and cyclin D2 gain-of-function mutations (microcephaly vs megalencephaly, respectively) are remarkably predictable considering that the two proteins heterodimerize to produce an active protein kinase. It suggests that CDK6 and cyclin D2 are faithful partners in the control of neural progenitor cell division, which is not always observed with other CDK/cyclin pairs controlling other mechanisms. In direct support of this hypothesis, cyclins D1 and D2 show both overlapping and distinct expression patterns during mouse forebrain development, which indicates that both cyclins exert unique functions in neurogenesis [45].

### Cyclin M and STAR syndrome

A novel developmental disorder was identified in four unrelated girls and in a previously reported mother-daughter pair [29, 46]. Because the cardinal features of the syndrome include toe syndactily, telecanthus and anogenital and renal malformations, the disorder was dubbed “STAR syndrome”. All individuals suffer from growth retardation, and additional anomalies affecting heart, eyes and/or cranial bones are observed in some of them. All six cases present heterozygous deletions or point mutations affecting the *FAM58A* gene, located on the X-chromosome. Over the past decade, very few other STAR syndrome patients have been reported, some of them presenting additional features such as hearing loss, multiple ocular defects, tethered spinal cord, skeletal anomalies, or lax joints [30–34]. The only lethal form reported so far included yet additional anomalies such as cleft lip palate, abdominal wall defect and cerebral malformations [35]. Since most of these additional features, including those seen in the lethal case, are associated to large deletions that extend beyond the *FAM58A* locus, the loss of neighboring genes is suspected to contribute to these expanded phenotypes [29, 31, 35]. All mutations or deletions appear to be sporadic except for the four mother-daughter pairs identified so far [29, 32–34]. In one of these pairs, maternal mosaicism with a minor toe syndactily phenotype was detected, which indicates that gonadal mosaicism is possible [34]. Total or close-to-total X-inactivation skewing was observed whenever it was investigated, thus suggesting that cells carrying the mutation on the active X chromosome present a growth disadvantage during development. This finding is congruent with the fact that STAR syndrome has never been found to affect males.

The functions of the *FAM58A* gene were totally unknown when STAR syndrome was first described. A few years later, the *FAM58A* gene product was shown to be the activating cyclin (then named cyclin M) of CDK10, which was standing out as one of the last orphan cyclin-dependent kinases, with no identified regulatory subunit. The CDK10/CycM heterodimer is an active protein kinase that phosphorylates the ETS2 oncoprotein, a previously identified binding partner of CDK10. Importantly, the truncated cyclin M proteins that might be expressed from two STAR *FAM58A* mutated forms are unable to bind CDK10 [47]. Since the majority of the other allelic variants consist of large deletions or splice site mutations likely to prevent cyclin M expression, it can be concluded that the CDK10/CycM protein kinase is deficient in STAR syndrome. The phosphorylation of ETS2 by CDK10/CycM positively controls ETS2 degradation by the proteasome. As expected, STAR patient-derived cells present higher ETS2 protein levels than control cells [47]. Interestingly, some morphologic features of STAR syndrome patients might be caused by abnormally high ETS2 levels, which, in *Ets2* transgenic mice, cause a number of cranial and skeletal defects [48]. It was later shown that the silencing of cyclin M, or that of CDK10, promotes assembly and elongation of primary cilia. These experimental findings combined with the observation of abnormal, elongated cilia on a STAR patient renal biopsy, strongly suggest that STAR syndrome is yet another ciliopathy [49].

### CDK10 and Al Kaissi syndrome

Nine individuals from five families presenting growth retardation, spine malformation, facial dysmorphisms, developmental delay and intellectual disability were investigated and shown to harbor homozygous mutations in the *CDK10* gene [10]. All mutations result in frameshifts or internal truncations that reduce CDK10 levels probably through NMD of the mRNAs. This medical condition can thus be attributed to a loss-of-function of CDK10. Accordingly, enhanced ETS2 protein levels are detected in patient fibroblasts and a mouse *CDK10* knockout model partly replicates the phenotype observed in the patients, particularly skeletal defects [10]. Here again, as could be expected, these skeletal defects are reminiscent of those of *ETS2* transgenic mice [48]. Simultaneously, another study reported one patient with globally similar defects and additional features, attributed to a homozygous single nucleotide deletion in the 11th of the 13 exons of *CDK10* [11]. In contrast to the above-described cases, the mRNA does not undergo NMD and it even appears to be slightly upregulated. Unexpectedly, patient fibroblasts present shorter, less abundant primary cilia [11], whereas RNAi-mediated CDK10 silencing in a human cell line or *CDK10* knockout mouse embryonic fibroblasts exhibit longer, more abundant cilia [10, 49]. This reported mutation results in a frameshift that might allow the translation of a shorter CDK10 protein (307 amino acids vs 360 for the longest wild-type isoform), containing 17 missense amino acids at its C-terminus. If expressed, this truncated CDK10 would be devoid of the C-terminal bipartite nuclear localization sequence present in the wild-type protein [50]. It thus might keep some of its extranuclear functions, among which is the regulation of actin dynamics and ciliogenesis [49]. It is also worth noting that this mutated, recessive allele is found in 36 of the 141,000 healthy individuals whose genomes have been deposited on the gnomAD database [51]. Almost all carriers are of Ashkenazi-Jewish descent, which represents a remarkably high carrier rate of 1/290 in this community.

The significant differences between the STAR and the Al Kaissi syndromes suggest that CDK10 and/or cyclin M exert more functions than those exerted by the CDK10/CycM protein kinase. However, as previously observed with other CDKs and cyclins, it is also possible that other members of these two protein families (such as cyclin G2 [52]) partially compensate the loss of CDK10 or cyclin M to fulfill at least some of the functions of the CDK10/CycM protein kinase.

### CDK13 and a congenital heart defect, craniofacial and intellectual development syndrome

CDK13 was associated to a developmental disorder by two recent exome-wide studies on large patient populations. First, the exomes of < 1900 patients suffering from syndromic and nonsyndromic congenital heart defects were sequenced and analyzed, in order to identify and characterize damaging de novo mutations (DNMs). 12 genes with genome-wide significance were shown to present an excess of DNMs, among which was *CDK13* [12]. Second, pursuing the same objective, the exomes of 4300 families containing individuals suffering from developmental disorders, and those of another 3300 individuals with similar problems were analyzed. Ninety-four genes were found significantly enriched in DNMs, among which was CDK13 again, the only CDK / cyclin family member identified in this study [13]. To date, a total of 52 patients carrying heterozygous mutations in *CDK13* have been reported [12, 14–20]. Their cardinal features include developmental delay, craniofacial defects, intellectual disability, feeding problems and, in half of the cases, structural brain and heart anomalies. A thorough clinical delineation of the syndrome has been reviewed recently [18, 53]. In the last reported case [19], the patient presented with pseudohypoaldosteronism, a disorder characterized by salt wasting resulting from target organ unresponsiveness to mineralocorticoids. It remains to be determined whether this additional feature is coincidental or attributable to the mutation in *CDK13*.

With more than 1500 amino acids, CDK13 is an unusually large CDK that contains an expanded serine-arginine (SR)-rich region in its N-terminal part, in addition to the central protein kinase domain. As expected from a SR domain-containing protein, CDK13 is involved in pre-mRNA splicing regulation and interacts with other splicing factors [54]. Its regulatory subunit is cyclin K [55]. The CDK13/CycK protein kinase, akin to other so-called transcriptional CDKs, phosphorylates the C-terminal domain (CTD) of RNA polymerase II and contributes to the control of gene expression [56]. The vast majority of the clinical cases reported so far (41/52) present missense mutations affecting the kinase domain, more than half of which targeting the highly conserved asparagine 842. One case presents a missense mutation upstream of the kinase domain, three cases present splice site mutations in the kinase domain, two cases present nonsense mutations in the C-terminal extension of the kinase domain, and five cases present a frameshift or a nonsense mutation that truncate the protein upstream of the kinase domain (reviewed in [53]). The functional consequences of these mutations remain uncertain. Simple haploinsufficiency resulting from an impaired kinase activity seems unlikely, since a number of loss-of-function mutations have been identified in the gnomAD database that excludes genomes of individuals suffering from severe pediatric diseases. Based on the crystallographic structure of the CDK13/CycK complex [56], the modeling of a number of missense variants and of one of the two splice mutants predicts a preserved ability to interact with cyclin K and a likely loss of catalytic activity [16]. Such variants would act as dominant negative mutants by sequestering cyclin K from the CDK13 wild-type allele, a well-described inhibitory mechanism for CDKs [57]. However, CDK13 haploinsufficiency might contribute to the disease in the 5 cases that present truncating mutations upstream of the kinase domain [18, 20].

### Cyclin K and a neurodevelopmental disorder/facial dysmorphism syndrome

Recently, a new syndromic neurodevelopmental disorder with facial dysmorphism was described in four unrelated individuals, who harbor de novo heterozygous changes affecting *CCNK* (cyclin K coding gene) [23]. Three patients present specific deletions in the 14q32.3 region, which overlap on three genes in addition to *CCNK*. Statistical considerations designated *CCNK* as the prime suspect, and its involvement was further established by the finding of a missense mutation (K111E) in a fourth individual with similar phenotypic presentations. These include impaired intellectual, motor, language skills and a constellation of facial dysmorphisms. The mutated residue being located at the interaction interfaces with CDK13 [56] and CDK12 [58], the amino acid substitution is expected to destabilize both complexes and hence to inhibit both kinases [23]. Haploinsufficiency is thus the most likely pathogenic mechanism in all four patients.

Little is known on the roles of CDK13 and cyclin K in development. In mouse, cyclin K-dependent protein kinases maintain self-renewal of embryonic stem cells [59] and CDK13 (and 12) positively regulate axonal elongation by controlling CDK5 expression [60]. *CNNK* knockdown and knockout experiments in zebrafish produce some dismorphic features that are reminiscent of those observed in the four patients, thereby confirming a role of cyclin K in neurodevelopment [23]. As for the syndromes caused by mutations affecting cyclin M and CDK10, the only partial phenotypic overlap between the syndromes caused by *CDK13* and *CCNK* mutations can be explained by the fact that at least two different protein kinases (CDK13 and CDK12) are affected by *CCNK* haploinsufficiency. However, the suspected capacity of many CDK13 pathogenic mutants to sequester cyclin K in inactive complexes would predict a dominant negative effect on both CDK13 and CDK12 kinases. Another explanation could be that cyclin K exerts functions independently from CDK12 or CDK13, and mingles with other CDKs such as CDK9 [61].

### Cyclin O and congenital mucociliary clearance disorder

Multiple motile cilia (MMC) are present on a number of epithelia (such as those found in lungs) and their beat plays a crucial role in clearing airways from debris, particles and microbes. Reduced ciliary motility results in mucociliary clearance disorders such as cystic fibrosis and primary ciliary dyskinesia (PCD) (reviewed in [62]). A new type of mucociliary clearance disorder was reported in 16 individuals from different consanguineous families, suffering from recurrent respiratory symptoms (upper and lower airway infections, evolutive damages and thickening of bronchial tubes). Whole-exome sequencing revealed seven different homozygous mutations occurring in the 3 exons of the *CCNO* gene that codes for cyclin O. Fifteen of the 16 patients present mutations that predict truncated forms the protein (88 to 321 amino acids out of 350 for the wild-type), if they are expressed. One patient presents a missense mutation affecting the highly conserved histine 239 residue [24]. Additional cases and mutations were reported subsequently, including a missense mutation affecting leucine 213, another highly conserved residue [25–28]. Respiratory epithelial patient cells show complete absence or severe reduction of cilia numbers and a marked decrease of basal bodies, attributed to an improper amplification and migration of the centrioles that nucleate ciliary axonemes [24, 26]. None of the putative truncated forms of cyclin O could be detected in patient cells (including a 321 amino acid-long theoretical variant) [24]. Moreover, contrary to wild-type cyclin O, the pathogenic L213P mutated form does not increase the number of basal bodies when expressed in Xenopus skin cells [26]. All mutations are thus considered loss-of-function. Cyclin O expression is detected in the apical cytoplasm of respiratory epithelial cells, which supports a role in mother centriole amplification [24]. It is also detected in murine epithelial cells of other multiciliated tissues such as ependymal cells in the developing brain and the fallopian tubes of juvenile and adult mice, with no apical localization in the latter [26]. This strongly suggests that cyclin O participates in the generation of MMCs during development, possibly in association with a still unidentified CDK.

### CDK19 / CDK8 and syndromic developmental disorders with intellectual disability

A single female patient was investigated for presenting with bilateral congenital retinal folds, microcephaly, scattered café-au-lait skin pigmentations, moderate psychomotor retardation and hearing loss. Karyotyping revealed a de novo heterozygous pericentric inversion in chromosome 6, and comparative genomic hybridization ruled out large copy number anomalies. The breakpoints were precisely mapped and one of them was found to affect intron 1 of *CDK19*, whose transcript level in patient-derived cells was found to be half of that of control cells. Since no anomaly was detected in the second copy of the gene, the patient’s condition is most likely caused by a *CDK19* haploinsufficiency [21].

More recently, 8 heterozygous missense mutations in *CDK8* were found by whole-genome or whole-exome sequencing in 1 and 11 patients enrolled in a craniosynostosis and a developmental disorder cohort, respectively. Their phenotypic presentations are variable and complex. Mild to moderate developmental delay is a universally shared feature. Facial dysmorphisms, hypotonia causing motor delays, and behavioral symptoms such as autism spectrum disorder and/or attention deficit hyperactivity are very frequently observed. Other diverse defects are observed less frequently: agenesis or thinning of corpus callosum, sensorineural hearing loss, ophtalmological and/or ano-rectal abnormalities, congenital heart defects. Only one patient presents craniosynostosis [9]. Seven mutations were detected only once, whereas the S62L variant was present in 5 patients. None of these mutations were found in the common variation databases and they arose de novo in the 10 cases whose paternity could be confirmed and analyzed [9].

The molecular and structural consequences of the 8 detected mutations were carefully examined and discussed [9]. They all cluster within the kinase domain, around the ATP binding pocket. None of them cause major protein instability and all mutants retain the ability to bind ATP and cyclin C. However, they all show a partially reduced kinase activity on the STAT1 substrate when expressed in a CDK8^−/−^/CDK19^−/−^ cell line. Although all these observations would point haploinsufficiency as being the pathogenic mechanism, the absence of truncation-inducing mutations or deletions is quite surprising, since these defects usually account for more than half of the haploinsufficiencies detected in developmental disorders [13]. Moreover, *CDK8* is moderately constrained to loss of function and it presents 6 bona fide truncating alleles listed in the gnomAD database, suggesting that heterozygous, truncated loss-of-function *CDK8* variants occur at low frequency in healthy individuals. These considerations strongly argue against haploinsufficiency and rather support a dominant-negative activity, assuming that an only partial loss of CDK8 activity can produce important effects during development. The heterogeneity of the phenotypic presentations cannot be easily linked to the different missense mutations, and it might stem from minor variations in the residual levels of CDK8 activity.

CDK8 and CDK19 are highly similar CDKs (> 90% sequence homology) that both interact with cyclin C to form two distinct mediator kinase modules. Mediator of RNA polymerase II is an evolutionary conserved multisubunit protein complex that bridges transcription factors to the basal transcriptional machinery (Pol II and the TFII general transcription factors), thereby controlling the assembly of the preinitiation complex on gene promoters. The up to 30 protein components of Mediator form 4 distinct modules, among which is the CDK kinase module, containing either CDK8/CycC or CDK19/CycC, associated to MED12 and MED13 or MED12L and MED13L, respectively. Phosphorylation of Mediator subunits, transcription factors and components of the basal transcriptional machinery plays a pivotal role in transcriptional regulation, and CDK8/CDK19 take their part together with other CDKs and other kinases (reviewed in [63]). CDK8 is known to regulate a number of signaling pathways that play important roles during development (such as Notch, Wnt/β-catenin, Sonic Hedgehog) (reviewed in [64]), which can explain the constellation of defects produced by CDK8 mutations. *CDK19* transcripts are highly expressed in fetal eye and fetal brain human tissues [21]. However, the role of the CDK19 kinase module during development remains poorly understood and is worth exploring in light of the human phenotype caused by a haploinsufficiency.

### Human developmental syndromes reveal CDK/cyclin functions

For a number of CDKs and cyclins involved in the developmental disorders reviewed above, the pathological phenotypes could be more or less accurately predicted from the biological knowledge that has accumulated for up to 25 years, sometimes with the help of mice knockout models. An excellent example is provided by CDK5, whose pivotal role in the development of the central nervous system has been well documented [38]. *CDK5* mice knockouts present an abnormal corticogenesis with lack of neuronal lamination and a cerebellar hypoplasia, as observed in CDK5 mutated patients (reviewed in [36]). Another example is cyclin D2, whose role in neuronal development has been identified [2] and whose mouse knockout models show a loss of cortical intermediate progenitor cells, laminar thinning and microcephaly [65], in congruence with the megalencephaly caused by gain-of-function mutations in humans.

However, in most cases reviewed herein, human phenotypes have provided novel, important information on the functions of CDK or cyclin genes targeted by pathogenic mutations. The fact that STAR syndrome presents features that are frequently observed in ciliopathies, such as renal, retinal and digital anomalies, prompted the exploration of a putative role of CDK10/CycM in ciliogenesis. This led to the demonstration that this protein kinase promotes assembly and elongation of primary cilia through the disruption of the actin network involving a PKN2-RhoA regulatory axis [49]. Without STAR syndrome, the role of CDK10/CycM in ciliogenesis would probably be still unknown, especially if one considers that CDK10 was not retained among the hits of a sub-genome-scale RNAi screening that identified a number of novel modulators of ciliogenesis and cilium length [66]. Another remarkable illustration of the contribution of human genetics to fundamental knowledge concerns cyclin O, whose functions were almost totally obscure until its involvement in a mucociliary clearance disorder was unveiled [24]. Studying this syndrome allowed to discover that cyclin O drives the formation of MMCs during development by controlling the proper formation of deuterostomes and the amplification of centrioles. This role was subsequently confirmed by *CCNO* knockdown in Xenopus [24] and knockout in mice [67], where cyclin O deficiency was shown to compromise deuterosome-mediated generation of MMCs in multiciliated cells. Another kind of case study is CDK6, which, akin to all cell cycle CDKs, has been quite extensively studied for more than two decades and is even one of the targets of the first clinically approved CDK inhibitors [4]. Yet, it is the finding that CDK6 mutations cause a MCPH syndrome that led to the discovery of the localization of CDK6 in centrosomes of dividing cells. The suggested novel role in centrosome function was confirmed by RNAi-mediated knockdown experiments, and patient-derived and CDK6 knowkdown cells were then shown to present reduced motility and polarity [8].

### CDK/cyclin human disorders are variably phenocopied by mouse models

In line with the tremendous interest that CDKs and cyclins have attracted, animal models and in particular mouse knockouts have been produced for most members of both families. As presented above, such models have proven very useful in deciphering the functions of some CDKs and cyclins, and they have sometimes offered valuable phenocopies of human syndromes caused by loss-of-function mutations. However, in other cases, animal models have partially or totally failed to do so. For example, *Cdk10* KO mice phenocopy the growth retardation and the spine defects observed in CDK10-mutated patients, but they present many additional defects in lung, kidney, heart, spleen, liver, muscle that are not seen in patients and that cause massive lethality before or soon after birth [10]. Whereas *CCNK* haploinsufficiency causes a number of human anatomical and functional phenotypes that should be potentially detectable in mice, *Ccnk*^+/−^ pups and adults are perfectly healthy and reproduce well [55]. Whereas respiratory problems represent the cardinal feature of patients containing homozygous loss-of-function *CCNO* mutations, none of those conditional KO *Ccno*^−/−^ mice that survived gestation presented such problems. However, *Ccno* conditional and constitutive KO mice present hydrocephalus and/or infertility [67, 68], two features occasionally observed in cyclin O-deficient patients [26, 69]. Whereas heterozygote *Cdk8*^+/−^ mice show no phenotype, it is impossible to obtain *Cdk8*^−/−^ offspring or even late embryos [70]. *CDK19* KO mice seem to show no significant functional or behavioral phenotype except for increased grip strength in males (www.mousephenotype.org).

Many reasons can explain the frequent inability of mouse KO models to phenocopy human disorders caused by mutations on a number of CDK or cyclin genes. First, it is well known that functional redundancies exist amongst the CDK and, even more so, the cyclin family. Hence, single gene KOs often produce no or modest phenotypes due to compensatory mechanisms (reviewed in [71]). Such redundancies and compensatory mechanisms also exist in humans. However, despite tremendous progresses [72], mouse phenotyping does not equal human full clinical investigations, and many human phenotypes (especially intellectual or behavioral abilities) can hardly be detected in mice. Second, mouse KO models only have the potential to phenocopy human syndromes caused by loss-of-function mutations. A number of syndromes reviewed herein are caused by gain-of-function or dominant-negative mutations, which, in the latter case, can conceivably preserve some of the functions of the corresponding protein. With the recent advances in genome editing techniques, it is now possible to introduce those mutations in mice to model human pathological conditions caused by gain-of function or dominant-negative mutations [73].

### Human genetics looms CDK/cyclin regulatory networks

Mutations on different loci often produce overlapping human phenotypes or, in some cases, a single given syndrome. This frequently reflects physical and/or functional interactions formed between the corresponding proteins to control underlying biological mechanisms. An emblematic example is the Bardet-Biedl ciliopathy that can be caused by mutations on more than 20 so-called BBS genes. Eight of these genes code for proteins forming an octameric complex (the BBsome), which plays a pivotal role in ciliogenesis [74]. Another one is provided by a number of syndromes that all include neurodevelopmental disorders and that are caused by mutations on components of the mediator of RNA polymerase II. In particular, 5 of the 7 proteins that form the kinase module, among which CDK8 and CDK19, are involved in these *mediatorpathies* [9, 75]. As highlighted by the few chosen examples below, the involvement of CDK and cyclin genes in human syndromes can point to unknown protein interactions or can give weigh to putative protein interactions identified by high-throughput proteome-scale endeavors, within a number of regulatory networks.

**Fig. 1:**
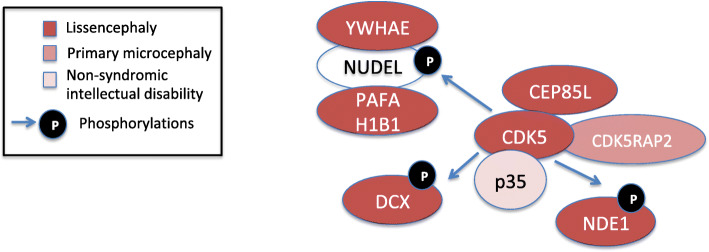
CDK5-centered regulatory network and its involvement in human neurodevelopmental disorders. PAFAH1B1 (aka LIS1) and YWHAE bind to CDK5/p35-phosphorylated NUDEL, thereby protecting it from degradation. Two other proteins involved in lissencephalic syndromes, DCX and NDE1 (aka LISX1 and LIS4, respectively), are also phosphorylated and regulated by CDK5, which thus plays a central role in the neuronal development mechanisms underlying lissencephaly

Most of the 20 genes involved in lissencephaly are related to microtubule structural or associated-proteins [36]. CDK5 was an already well-known regulator of neuronal cytoskeleton dynamics, directly or indirectly engaging some of these 20 proteins (reviewed in [76]). The recent discovery of a new lissencephalic syndrome caused by mutations in *CEP85L* allowed to unveil an important interaction with CDK5, which drives the localization and activation of the kinase at the centrosome to ensure proper organization of centrosome and cytoskeleton [77] (Fig. 1).

**Fig. 2:**
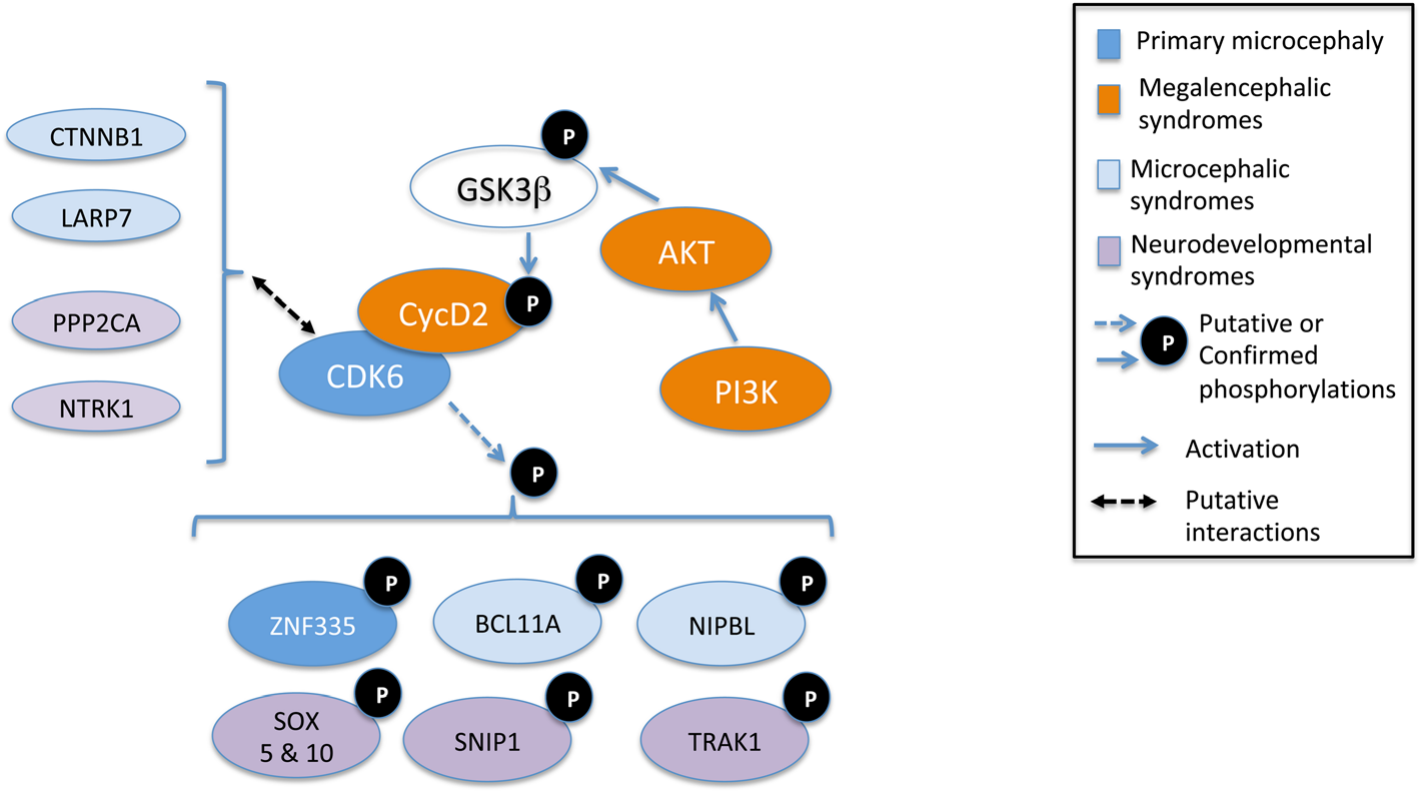
CDK6/CycD2-centered regulatory network and its involvement in human neurodevelopmental disorders. Cyclin D2 stabilization causes MPPH and can be caused by mutations in the *CCND2* gene or in the genes coding for AKT or for the PI3K subunits, which result in GSK3β phosphorylation and inhibition. A number of CDK6 putative phosphorylation substrates and putative interacting partners are involved in MCPH, other microcephalic syndromes, or other neurodevelopmental syndromes. Data sources: the Online Mendelian Inheritance in Man database (www.omim.org) and the Biological General Repository for Interaction Datasets (www.thebiogrid.org)

Autosomal recessive primary microcephaly can be caused by mutations on 25 different so-called MCPH genes and a large proportion of them, including *CDK6*, regulate centrosomes and mitotic spindle [40]. A systematic effort to identify CDK4/6 phosphorylation substrates revealed that MCPH10 (aka ZNF335) is phosphorylated by CDK6/CycD in vitro [78]. BCL11A, another identified zinc finger phosphorylation substrate, is involved in the Dias-Logan syndrome that includes microcephaly among other features [79]. Interestingly, in mice, *Bcl11a* deficiency was shown to downregulate *Cdk6* [80], which strengthens the hypothesis of a functional interplay between both proteins. Other CDK6/CycD in vitro phosphorylation substrates include SNIP1, SOX10, TRAK1 and SOX5, which are all involved in different human neurodevelopmental disorders, and which should thus be also considered as prime candidate substrates of functional significance. Moreover, 4 of the 168 CDK6 interactors listed in the Biogrid database are involved in human disorders that include microcephaly. Although this could result from a purely random coincidence, these candidate interactors, which were mostly identified by high-throughput approaches, should be considered with higher scrutiny. MPPH syndromes can be caused by activating mutations in the genes coding for AKT3 or for the regulatory or catalytic subunit of PI3K [81]. Activation of the PI3K-AKT pathway is known to inhibit GSK-3β, a protein kinase that phosphorylates and tags cyclin D2 for degradation [44]. As could be expected, cyclin D2 is stabilized in cells derived from MPPH patients with activating mutations on the PI3K-AKT axis. This strongly points cyclin D2 stabilization as the shared endpoint of the MPPH syndromes (Fig. 2).

STAR syndrome presents a strong clinical overlap with Townes-Brocks syndrome (TBS), so much so that some STAR patients have been initially diagnosed with TBS [32]. Because TBS is caused by mutations on the transcriptional repressor SALL1 [82], it was hypothesized and demonstrated that cyclin M and SALL1 interact, akin to their respective paralogs cyclin D1 and SALL4 [29]. The function of this interaction remains to be determined.

**Fig. 3:**
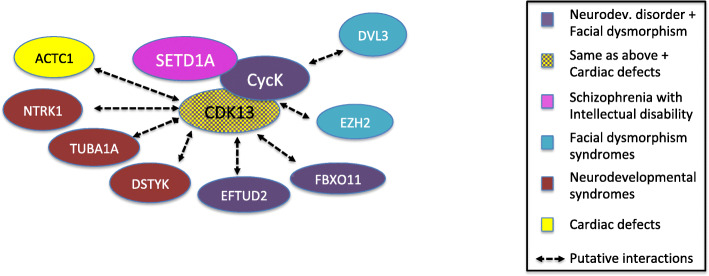
CDK13/CycK-centered regulatory network and its involvement in human developmental and behavioral disorders. Except for the CDK13-CycK and CycK-SETD1A interactions, all other interactions have been detected by high-throughput studies and are thus putative. EZH2 and DVL3 are involved in the Weaver and Robinow syndromes, respectively. Data sources: same as Fig. 2

Cyclin K was recently found to interact with the histone methyltransferase SETD1A that recruits it to the chromosomes, where it is required to ensure DNA damage response by controlling the expression of genes involved in DNA repair [83]. Interestingly, loss-of-function mutations in *SETD1A* are associated to schizophrenia and intellectual disabilities [84], the latter being observed in all CCNK-deficient patients. A number of high-throughput studies have identified putative CDK13 and/or cyclin K interacting partners, some of which are involved in human disorders that present overlapping phenotypes with the syndromes caused by loss-of-function mutations in *CDK13* and *CCNK* genes. For example, ACTC1 is involved in a number of heart defects, among which is atrial septal defect, frequently observed in CDK13-deficient patients [53]. Here again, these putative interactions should be further explored with more attention than others (Fig. 3).

### *Edgotyping* CDK and cyclin pathogenic variants

Missense mutations represent more than half of reported mutations that cause Mendelian disorders [85]. The previously mentioned large-scale analysis of DNMs causing developmental disorders revealed that 23% of the individuals carry missense or protein-truncating mutations [13]. Nonsense or frameshift mutations introduce premature termination codons, which, depending on their localization in the genes, can be insensitive to NMD and allow the expression of truncated proteins (reviewed in [86]). Missense or protein-truncating mutations can either compromise the structure of proteins, which are then usually degraded by the proteasome, or compromise specific interactions with other (macro)-molecules. A large-scale study on almost 3000 missense mutations concerning 1140 genes revealed that a majority of disease-causing mutations do not dramatically affect protein structure or folding. Interestingly, while common variants rarely affect interactions, two-thirds of pathogenic variants show perturbed protein interaction profiles, half of which presenting selective losses of interactions [87]. *Edgotyping* (i.e. determining the interaction profiles of) such “edgetic” variants can be extremely informative on pathogenic mechanisms and on the functions exerted by crucially important protein interactions. Among the plethora of protein interaction assays that have been developed, transcriptional yeast two-hybrid mating assays are particularly well suited for this purpose [88].

**Fig. 4:**
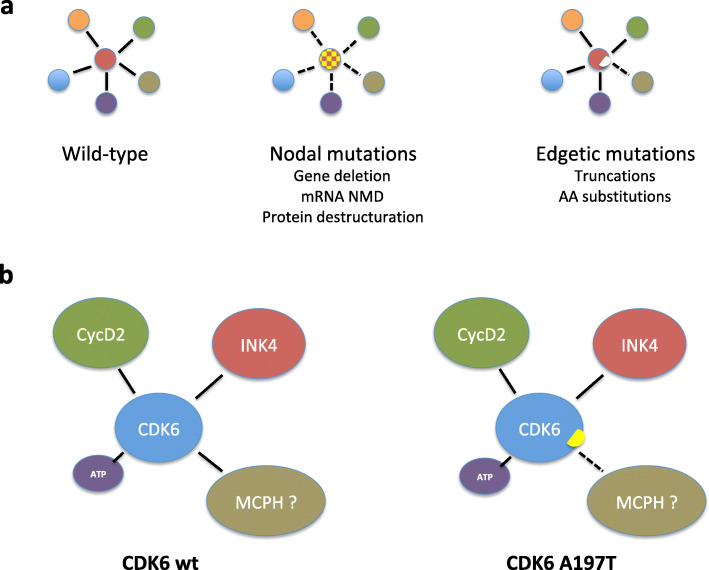
Edgotyping disease-causing protein variants. **a** left: a wild-type protein (red) interacts with 5 different proteins; middle: all interactions are lost because the protein is no longer expressed (gene deletion, nonsense or frameshift mutations causing mRNA NMD), or its structure is severely compromised (all kinds of mutations); right: only one of the interactions is lost (truncation-inducing or missense mutation). **b** Edgotyping of the CDK6 A197T variant, which is suspected to retain its enzymatic activity but which no longer localizes in the centrosomes because of a hypothetic loss of interaction with a centrosomal protein that remains to be identified

A number of CDK /cyclin mutations reviewed herein likely produce edgetic variants, which remain to be characterized. CDK13 pathogenic missense mutations in the kinase domain are thought to compromise CDK13 catalytic activity by causing loss of ATP binding while retaining its capacity to interact with cyclin K, thereby acting as dominant negative mutants [16]. However, a number of patients present protein-truncating mutations that occur in the 5′ half of the gene, which rather points to haploinsufficiency. Edgotyping the diverse set of pathogenic CDK13 variants should illuminate the molecular etiology of the syndrome. Likewise, such an effort would directly test the hypothesized loss of interaction between the cyclin K K111E pathogenic variant and CDK13 (and CDK12) [23]. Edgotyping the CDK6 A197T pathogenic variant should confirm its ability to retain the interactions with cyclin D and the INK inhibitor [8], and it might provide an explanation for the fact that it no longer localizes in centrosomes if a loss of interaction with a known centrosomal protein is detected (Fig. 4). The inability to detect expression of cyclin O pathogenic variants (and in particular that of a 321 AA truncated variant) [24] is unlikely due to mRNA NMD and might result from a compromised interaction that normally stabilizes the wild-type protein. Edgotyping these truncated cyclin O forms and the two reported missense variants (one of which being detectably expressed [26]) should prove very informative.

## Conclusion

Over the past decade, the harnessing of next-generation sequencing methods to understand human genetic diseases has considerably accelerated the discovery of pathological mutations. So far, mutations in 6 CDKs and 4 cyclins have been involved in rare human developmental disorders. Considering the importance and the variety of functions exerted by CDKs and cyclins in cellular processes that play crucial roles in development, mutations in additional members of both families will undoubtedly be discovered in other syndromes. As highlighted herein, some of these future discoveries will probably reveal new functions even for those CDKs and cyclins that have been thoroughly studied for the past 30 years. Crossing various data obtained from genome and proteome-scale endeavors, such as protein interaction databases, with existing and future human genetics knowledge will allow to further chart regulatory networks around CDK / cyclin complexes and other important regulators. These expanded regulatory networks will be fruitfully exploited to edgotype missense and truncated CDK / cyclin pathogenic variants, which will strengthen our knowledge on these proteins and illuminate pathogenic mechanisms. Finally, although most CDK / cyclin-related disorders stem from abnormal development, few of them (and especially those caused by gain-of-function mutations) might conceivably offer opportunities of therapeutic interventions to alleviate some symptoms, using increasingly selective CDK inhibitors that are currently and will be developed as drugs [89].

## Data Availability

Not applicable.

## Abbreviations

ATP: Adenosine triphosphate
BBS: Bardet-Biedl syndrome
CDK: Cyclin-dependent kinase
CTD: C-terminal domain
DNM: De novo mutations
gnomAD: Genome aggregation database
KO: Knockout
LCH: Lissencephaly with cerebellar hypoplasia
MCPH: Autosomal recessive primary microcephaly
MMC: Multiple motile cilia
MPPH: Megalencephaly-polymicrogyria-polydactyly-hydrocephaly
NMD: Non-sense mediated decay
NS-ID: Non-syndromic intellectual disability
PCD: Primary ciliary dyskinesia
RNAi: RNA interference
RNA pol II: RNA polymerase II
STAR: Syndactily telecanthus anorectal
TBS: Townes-Brocks syndrome
